# Retraction: Awareness of residents about kala-azar and its related practices in two endemic areas of Bangladesh

**DOI:** 10.1371/journal.pone.0270656

**Published:** 2022-06-23

**Authors:** The PLOS ONE Editors

Following the publication of this article [1], concerns were raised regarding the authorship and ownership of the presented data. The Northern University Bangladesh investigated the authorship and data ownership concerns, and informed PLOS that the data presented in this article belong to another researcher, who was not included as an author on this article and who did not provide permission for the data to be used by the authors or to be shared under an Open Access licence.

In light of the institutional finding, this article [1] does not adhere to the *PLOS ONE* Licences and Copyright policy, and the article is being retracted upon request from the data owner. The retracted *PLOS ONE* article was removed from the *PLOS ONE* website at the time of retraction and the removed contents are no longer offered under the Creative Commons Attribution License.

The authors either did not respond directly to the editorial decision to retract this article or could not be reached.
